# Structure and membrane interactions of the homodimeric antibiotic peptide homotarsinin

**DOI:** 10.1038/srep40854

**Published:** 2017-01-19

**Authors:** Rodrigo M. Verly, Jarbas M. Resende, Eduardo F. C. Junior, Mariana T. Q. de Magalhães, Carlos F. C. R. Guimarães, Victor H. O. Munhoz, Marcelo Porto Bemquerer, Fábio C. L. Almeida, Marcelo M. Santoro, Dorila Piló-Veloso, Burkhard Bechinger

**Affiliations:** 1 Departamento de Química Universidade Federal de Minas Gerais, P.O.Box 486, 31270-901 Belo Horizonte, MG, Brazil; 2 Departamento de Química Universidade Federal dos Vales do Jequitinhonha e Mucuri, 39100-000 Diamantina, MG, Brazil; 3 Departamento de Bioquímica e Imunologia, Universidade Federal de Minas Gerais, P.O.Box 486, 31270-901 Belo Horizonte, MG, Brazil; 4 Laboratório de Espectrometria de Massa, Empresa Brasileira de Pesquisa Agropecuária (EMBRAPA) - Recursos Genéticos e Biotecnologia, Estação Parque Biológico, Final W5, Asa Norte, Brasília, DF, 70770-900, Brazil; 5 Centro Nacional de Ressonância Magnética Nuclear Jiri Jonas – CNRMN, Instituto de Bioquimica Médica, Programa de Biologia Estrutural, Universidade Federal do Rio de Janeiro, Rio de Janeiro, Brazil; 6 Université de Strasbourg/CNRS, UMR7177, Institut de Chimie, Strasbourg, France

## Abstract

Antimicrobial peptides (AMPs) from amphibian skin are valuable template structures to find new treatments against bacterial infections. This work describes for the first time the structure and membrane interactions of a homodimeric AMP. Homotarsinin, which was found in *Phyllomedusa tarsius* anurans, consists of two identical cystine-linked polypeptide chains each of 24 amino acid residues. The high-resolution structures of the monomeric and dimeric peptides were determined in aqueous buffers. The dimer exhibits a tightly packed coiled coil three-dimensional structure, keeping the hydrophobic residues screened from the aqueous environment. An overall cationic surface of the dimer assures enhanced interactions with negatively charged membranes. An extensive set of biophysical data allowed us to establish structure-function correlations with antimicrobial assays against *Gram*-positive and *Gram*-negative bacteria. Although both peptides present considerable antimicrobial activity, the dimer is significantly more effective in both antibacterial and membrane biophysical assays.

The granular glands (serous and poison glands) of many amphibians produce a variety of substances, including biogenic amines, peptides, steroids, and alkaloids^1^,^2^. Among peptides, a great number of cationic amphipathic structures have been described with membrane disrupting properties^3^ in many cases leading to cell disruption and death^4^,^5^,^6^. Once inside the cells they may also operate through interactions on intracellular targets and interfere with key cellular processes^3^,^7^,^8^.

Hundreds of naturally occurring antimicrobial peptides differing in length, amino acid composition, primary structure, hydrophobicity, amphipathicity and membrane-bound conformation have been isolated from amphibians^9^,^10^,^11^,^12^. They have been classified for shared properties that appear to be important for their structure and activity such as their cationic amphipathic character, regions rich in hydrophobic and aromatic residues^13^,^14^ or intra-chain cystine bonds as described for defensins^15^ and protegrins^16^. Whereas a good number of sequences carrying such intra-chain cystine bridges have been described^15^,^16^,^17^, dimeric peptides with inter-chain disulfide bonds are less common with the heterodimeric peptide distinctin being the only example published so far^18^. This peptide was discovered in amphibians and the covalent association between the two chains has been suggested to be an essential feature to improve its resistance to proteolysis^18^. Furthermore, the homodimeric peptide LLP1 has been created by design^19^. This sequence shows a significant increase in antimicrobial activity when the two chains are linked by a central disulfide bond^19^. In contrast, the ability of another peptide Ctx-Ha to inhibit the growth of microorganisms is reduced upon dimerization while its hemolytic activity increased^20^.

Here we present physico-chemcial investigations of a new homodimeric peptide named homotarsinin (Htr), which has originally been isolated from the skin secretion of *Phyllomedusa tarsius*, a green tree-frog inhabiting the Amazonian basin. Homotarsinin is composed of two equivalent chains, each containing 24 residues and carries a natural C-terminal carboxyamidation (NLVSD IIGSK KHMEK LISII KKCR-NH_2_) (Prates, M.V. and Bloch, Jr C., unpublished results). To our knowledge this is the first naturally occurring homodimeric membrane-active antimicrobial peptide described in the literature. Here, we have investigated the activity, the structure in aqueous environments as well as its membrane interactions in quantitative detail by a large variety of physico-chemical methods. In order to analyze the effect of the inter-chain interactions on the structural stability of the dimer, both the homodimer peptide (Htr) and its monomeric chain (Htr-M) were systematically compared to each other. In order to confirm the absence of Htr-M dimerization during the experiments, some analyses have also been performed with Htr-M in the presence of DTT as well as with the derivative peptide [C23S]Htr-M, where Cys-23 was replaced by serine. Thereby, to our knowledge, this is the first structural and biophysical characterization of a homodimeric antimicrobial peptide in aqueous and membrane environments. This study not only provides insights into the mechanism of action of this particular antimicrobial peptide but also about dimeric sequences in general.

## Results

### Peptide Synthesis and Characterization

Htr-M and the derivative peptide [C23S]Htr-M were obtained by solid-phase peptide synthesis using the Fmoc strategy and the respective identities were confirmed by mass spectrometry. These peptides were purified by semi-preparative reverse phase HPLC and the purity was verified by analytical HPLC and by mass spectrometry (Supplementary Fig. 1). The homodimer was obtained by air oxidation of Htr-M in a Tris-HCl buffer solution (pH 8.0), and the dimerization process was followed by analytical HPLC injections (data not shown). The reaction was stopped after 72 h and the dimer was purified by semi-preparative reverse phase HPLC having its identity and purity been verified by analytical HPLC and mass spectrometry analyses. The reaction yield was approximately 60% to prepare the monomer and an overall yield of 51% was obtained for the dimer (i.e. dimer formation occurred very efficiently at a yield of 85%). The purified samples were used for biophysical and microbiological investigations.

### Conformational Analysis

**Figure 1:**
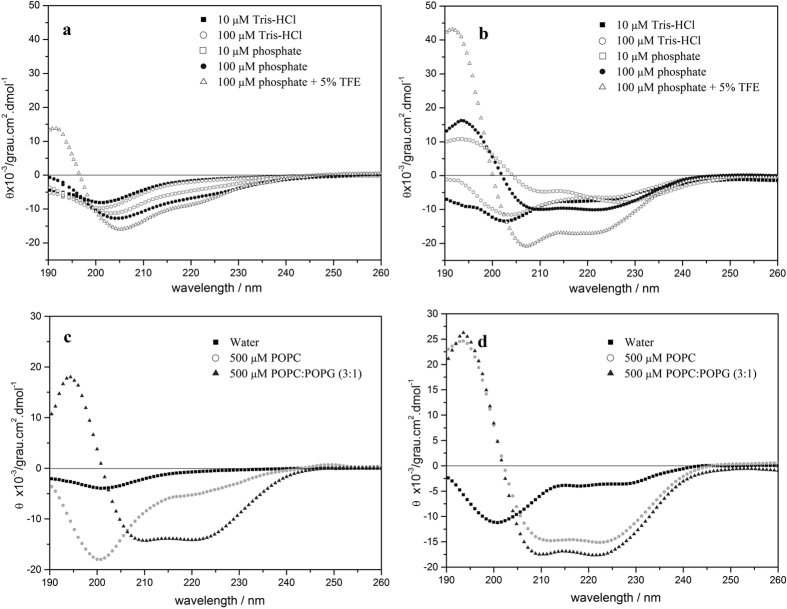
CD spectra of 49 μM Htr-M (**a**,**c**) and 51 μM Htr (**b,d**). (**a,b**) Spectra recorded for the peptides in aqueous buffer solutions: 10 μM Tris–HCl, pH 8.0 (filled square), 100 μM Tris–HCl, pH 8.0 (open circle), 10 μM phosphate buffer, pH 7.0 (open square), 100 μM phosphate buffer, pH 7.0 (filled circle) and 100 μM phosphate buffer, pH 7.0 containing 5% TFE (open triangle); (**c,d**) Spectra recorded for the peptides in water (filled square) and in the presence of 500 μM phospholipid POPC LUVs (open circle) and POPC:POPG (3:1 mole:mole) LUVs (filled triangle).

In a first step circular dichroism spectra were recorded for Htr, Htr-M and [C23S]Htr-M in aqueous buffer solutions, as well as in the presence of a range of membrane-mimetic solvents and phospholipid vesicles. CD spectroscopy (Fig. 1) indicates that both the homodimer and the monomeric chain adopt helical structures in the presence of zwitterionic and negatively charged phospholipid vesicles or in TFE/H_2_O mixtures. Although significant structural degrees are obtained for both peptides in the different environments, in all situations higher helical contents are observed for the homodimer in comparison with the individual chain. In Tris-HCl buffer both peptides showed a minimum signal at about 200 nm typical of predominantly random coil conformations^21^ (Fig. 1a,b). Interestingly, while Htr-M does not present any structural preference in aqueous phosphate buffer, the spectrum recorded for Htr in a 10 μM phosphate buffer solution exhibits an additional negative signal at 222 nm, which is indicative of helical secondary structures (Fig. 1b). Indeed, when the phosphate concentration is increased up to 100 μM, a well-defined helical profile becomes obvious with minima at 222 and 208 nm and a maximum at 193 nm. This result clearly indicates that inter-chain interactions play an important role in the stability of the homodimer three-dimensional structure. Both peptides increase in helical content in the presence of either POPC:POPG (3:1, mole/mole) or POPC vesicles (Fig. 1c,d). The calculated α-helical contents of the monomer and the dimer in all environments are listed in Table 1. Notably, under all experimental conditions investigated the dimer presents a higher α-helical content when compared to the monomer. The CD spectra obtained for Htr-M in the presence of DTT and for [C23S]Htr-M are very similar to the spectra obtained for Htr-M under similar conditions (Supplementary Fig. 2), which indicates that spontaneous dimerization does not occur during the experiments performed with Htr-M.

### NMR spectroscopy

CD spectroscopy indicates that Htr adopts α-helical conformations in either aqueous phosphate buffer or in membrane mimetic environments, whereas Htr-M forms helical secondary structures only under the latter conditions. Therefore, in order to obtain detailed information about the three-dimensional structures of these peptides, solution NMR spectra were recorded for Htr in 20 mM phosphate buffer containing 5% of TFE-*d*_2_, and for Htr-M in a H_2_O/TFE-*d*_2_ 70/30 (v/v), where the small amounts of TFE help to solubilize the peptides and at the same time to stabilize their intrinsic propensity for secondary structure formation (Table 1).

Once the characteristic amino acid spin systems were identified for both peptides from the TOCSY spectra, unequivocal assignments were obtained from sequential and some medium range NOE correlations (Supplementary Fig. 3). Sequential connectivities are observed through d_αN_(i, i + 1), d_βN_(i, i + 1) and d_NN_(i, i + 1)NOE correlations, which are graphically summarized in Supplementary Figure 4. Sequential and medium range NOE correlations ranging from L2 up to the terminal carboxamide are observed in the NOESY spectrum of the monomer, which indicates the existence of a helical segment encompassing all of these residues (Supplementary Figs 3a,b and 4a). For the homodimer in phosphate buffer the helix is slightly shorter involving only the residues between V3 and K22 (Supplementary Figs 3c,d and 4b). Both Htr and Htr-M exhibit high contents in helical secondary structure. Furthermore, the two chains within the dimer structure show a high degree of similarity. As a consequence, the TOCSY, ^1^H-^13^C-HSQC and ^1^H-^15^N-HSQC contour maps of Htr-M in TFE-*d*_2_/H_2_O and the corresponding spectra of Htr in phosphate buffer closely resemble each other, which facilitated the complete assignment of the Htr contour maps.

However, in ^1^H–^13^C HSQC spectra a different chemical shift is observed for the C_β_ of the cysteine residue in Htr-M (28.7 ppm) when compared to both C_β_ in the cystine residue of Htr (36.5 ppm; data not shown). These values are in agreement with studies, which show that C_β_ cysteine/cystine residues are highly sensitive to the oxidation state of sulfur^22^,^23^. This is important additional evidence that the disulfide bond of the dimer has been properly formed.

Notably, in the TOCSY spectrum of the homodimer more amino acid spin systems have been distinguished than expected from the 24 amino acid residues of the corresponding monomers. Clearly, the two chains are in different chemical environments due to an asymmetric arrangement within the homodimer structure or due to two conformations in slow exchange. At the same time the NOE distance constraints are indicative of closely related conformations of the two chains.

**Figure 2:**
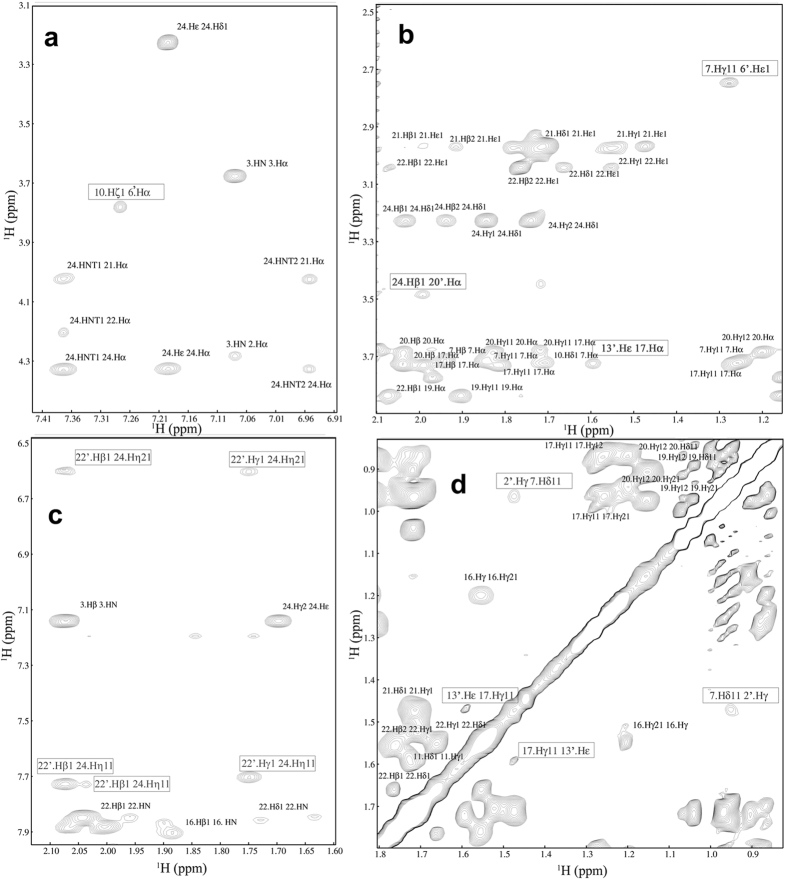
NOESY contour plot obtained for 1 mM Htr in 20 mM aqueous phosphate buffer containing 5% TFE-*d*_2_ (v:v). Boxed correlations correspond to long range inter-chain interactions.

A significant number of long range inter-chain correlations are also observed for the dimer, which suggests a tightly packed structural arrangement of Htr. Some of these correlations are boxed in expansions of the NOESY spectrum presented in Fig. 2. Close contacts are observed near the carboxamide- and the amino-termini as well as in the central portion of the dimer (*cf*. NOE correlations between R24 and K20′/K22′, and between I7 and L2′). Interestingly, an NOE correlation between K10′ NH_3ζ_ and the I6 hydrogens is also visible indicating that these often labile N-H bonds of lysine are stabilized in the dimer. Other long range correlations involving Arg-NH_η_ labile hydrogens (R24 NH_2η_ with K22′ H_γ_ and R24 H_η_ with K22′ NH_3ζ_) are also observed (Fig. 2).

Deuterium exchange experiments with the homodimer solubilized in a D_2_O:TFE-*d*_3_ (95:5) pH 7.0 phosphate buffer solution confirms a tight packing of the two helical segments within the homodimer coiled coil structure (Supplementary Fig. 9). On the other hand, deuterium exchange takes place almost instantly in aqueous environments in the absence of phosphate buffer, which is line with the CD data showing that the phosphate ions are essential to assure the helical conformation of the peptide in aqueous medium.

### Structure calculations

The structural statistics of Htr-M in TFE-*d*_2_/H_2_O and of Htr in phosphate buffer are presented in Table 2. When only the well-structured α-helical segments are considered in the ensemble of the twenty most stable structures, relatively small RMSD values are observed for both peptides, indicating a significant structural stability of these helical segments. When all residues are taken into account, slightly higher RMSD values are observed for both peptides, which is due to the lack of structural stability near the amino- and carboxamide termini. Finally, analysis of the Ramachandran plots shows that more than 94% of all residues are in the most favored regions of the diagram, indicating a high stereochemical quality of the twenty most stable structures obtained for both peptides. For the homodimer, the other residues are located in the additionally (4.8%) or in the generously allowed regions (0.6%). For the monomer, 0.5% of the residues are located in disallowed regions, however this small number is related to residues located near the amino-terminus, which is structurally not well defined.

**Figure 3:**
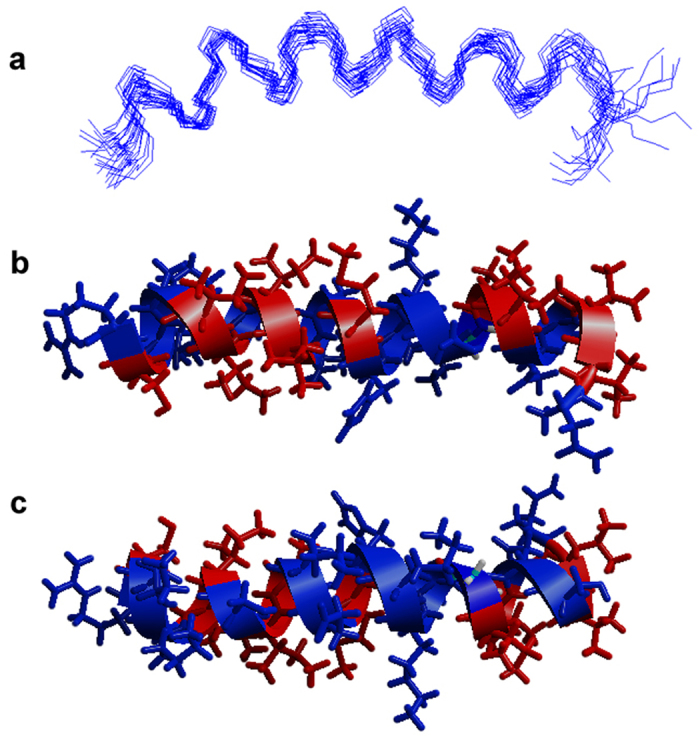
Solution NMR structure of 4 mM Htr-M in H_2_O/TFE-*d*_2_ 70/30 (v/v). (**a**) Superposition of the twenty lowest energy structures. The lowest energy structure viewed from the side of (**b**) the hydrophobic and (**c**) the hydrophilic helix face. The hydrophobic residues are represented in red and the hydrophilic residues in blue. The amino terminus is shown to the left.

**Figure 4:**
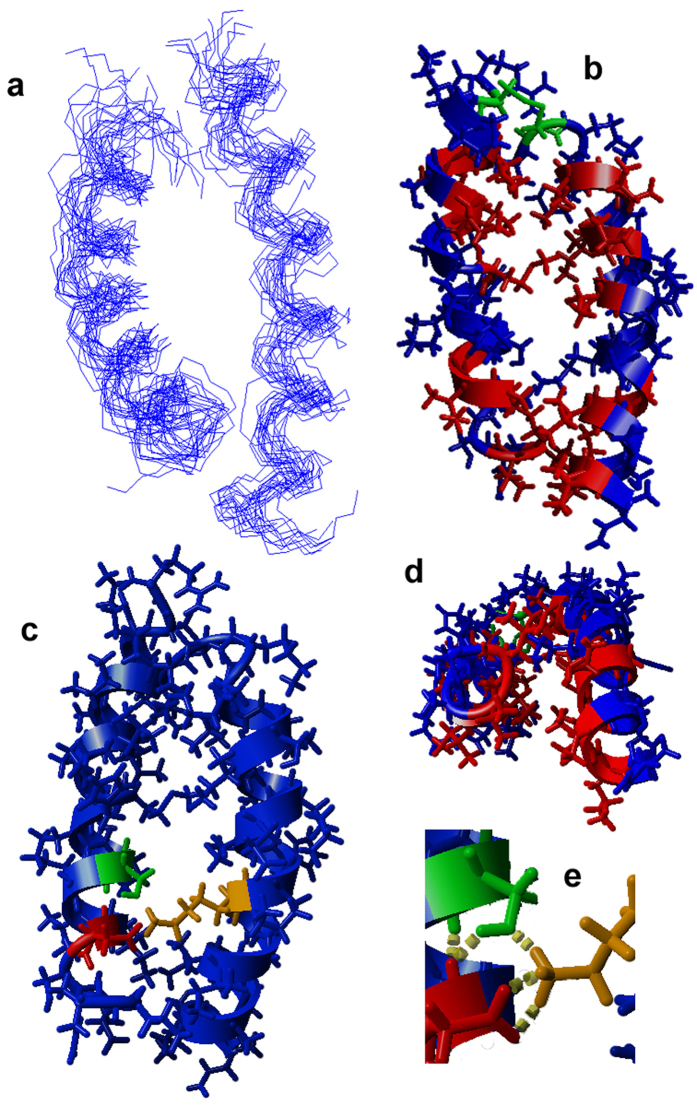
Solution NMR structure of 1 mM Htr in 20 mM aqueous phosphate buffer containing 5% of TFE-*d*_2_, (v:v). (**a**) Twenty lowest energy structures (superposition of residues 2–22 of chain 1 and 2′-22′ of chain 2). (**b,c**) The lowest energy structure viewed from the side and (**d**) along the coiled coil longitudinal axis. (**e**) View into the inner part of the coiled coil containing a hydrophilic cluster. In (**b)** and (**d)** the hydrophobic residues are represented in blue and the hydrophilic residues in red, while the cystine residue is represented in green. In (**c** and **e)** the hydrophilic residues involved in internal electrostatic interactions are highlighted (D-5 in red, S-9 in green and K-10′ in orange). The carboxy-terminus is shown on top (**a**–**c**) or in the back (**d**).

For both peptides, several sequential and medium range distance restrains allowed the determination of well-defined α-helical segments (Figs 3 and 4). Furthermore, long-range inter-chain NOEs are indicative of a well-defined coiled coil conformation of the dimer (Fig. 4b,c). Htr-M shows a significant amphipathic character, where the positively charged lysine residue (K10′) represents a discontinuity within the hydrophobic region. Therefore, Htr-M is not as amphipathic as other tree-frog peptides isolated from species of the *Phyllomedusinae* subfamily, such as the phylloseptins and DD K^24^,^25^. In the dimer, the hydrophobic residues form the core of the coiled coil structure, whereas the hydrophilic residues are exposed to the solvent molecules (Fig. 4b,d). The charged lysine residue (K10′) resides within the hydrophobic interior of the homodimer where it forms strong electrostatic inter-chain interactions with the negatively charged D5 and the polar S9 side chain (Fig. 4c,e).

### Size Exclusion Cromatography

In order to compare the oligomeric forms of homotarsinin and distinctin, size exclusion chromatography experiments were performed with both dimeric peptides at similar conditions (Supplementary Fig. 5). Distinctin eluted significantly faster than homotarsinin (retention times of 11.5 min and 22.8 min, respectively), although both dimers have very similar molecular masses.

### Isothermal titration calorimetry

**Figure 5:**
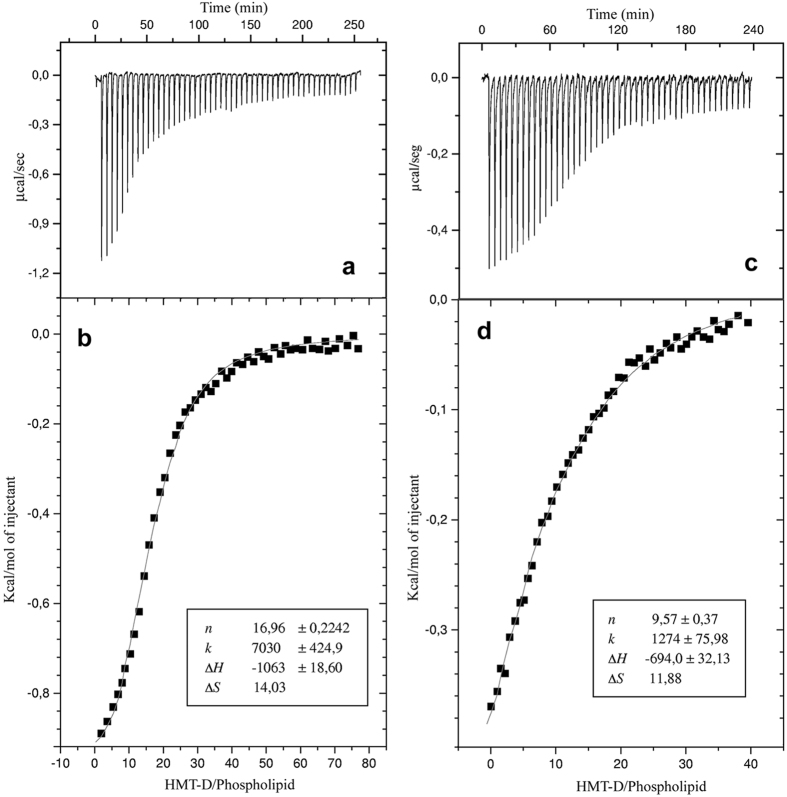
Isothermal titration calorimetry of 100 μM Htr in 10 mM Tris–HCl, pH 8.0, containing 200 mM NaCl with LUVs (20 mM stock solutions in 10 mM Tris–HCl, pH 8.0 containing 200 mM NaCl) made of (**a,b**) POPC:POPG (3:1) and (**c,d**) POPC. (**a,c**) The heat flow for each Htr injection as a function of time (raw data). (**b,d**) The enthalpy as a function of the Htr-to-phopholipid molar ratio. The baseline was corrected and the heat of dilution subtracted from each experimental point.

The membrane association of Htr, Htr-M and [C23S]Htr-M with POPC or POPC:POPG (3:1 mole/mole) LUVs was studied in a quantitative manner by isothermal titration calorimetry (ITC) (Fig. 5). Figures 5a and c present the titration of 10 mM POPC and POPC:POPG (3:1) LUVs, respectively, into a 100 μM solution of Htr. In Fig. 5b and d the heat of reaction is shown when the dimer associates with POPC or POPC:POPG (3:1) LUVs in the presence of Tris–HCl buffer. In both cases the major driving forces are related to entropic contributions, which exceed the enthalpic ones by four to five-fold (Supplementary Table 1). The binding isotherms have been determined by non-linear curve fitting using the model of equivalent binding sites (Fig. 5b,d). The magnitude of the interaction parameters obtained for Htr-M in the presence of phospholipid membranes are much lower than the values obtained for Htr under similar conditions and are of the same magnitude as the values obtained for the [C23S]Htr-M membrane interactions, suggesting that Htr-M does not under go dimerization during the titration experiments. The raw data (Supplementary Fig. 6) obtained for the assay of a 100 μM solution of [C23S]Htr-M and Htr-M in presence and absence of DTT titrated with 20 mM POPC:POPG (3:1) shows only moderate affinity (<1 × 10^3^ M^−1^)^26^. In contrast association of these peptides with POPC LUVs (results not shown) seems insignificant because the heats associated with injection of POPC LUVs into a solution of Htr-M or of [C23S]Htr-M cannot be distinguished from the heat of dilution when LUVs are injected into buffer. On the other hand, Htr presents a significant affinity to both phospholipid vesicles, with an association constant that falls within the range 10^3^–10^4^ M^−1^. The calculated thermodynamic parameters (Δ*G*°, Δ*H*°, Δ*S*°), the apparent binding constants *K*_*app*_, and the stoichiometric coefficients *n* are presented in Supplementary Table 1 for the interaction of the dimer with POPC and POPC:POPG (3:1) LUVs.

### Dye leakage measurements

The presence of Htr or Htr-M results in carboxyfluorescin release from POPC-LUVs or POPC:POPG-LUVs and was montored as a function of [Peptide]/[Phospholipid] molar ratio (Supplementary Figure 7). The CF leakage from LUVs without peptide addition is negligible during the time course of the experiment. The observed rate constants, *k*_obs_, of CF release and CF release vs. [Peptide]/[Phospholipid] ratio are presented in Supplementary Figure 7. In all cases the dye release increases with the addition of peptide, reaching a plateau at higher values. Notably, at a given time interval the CF release from either LUVs is always smaller for Htr-M when compared to Htr. Moreover, the homodimer shows a higher carboxyfluorescein release activity in the presence of Tris-HCl when compared to phosphate buffer.

### Dynamic Light Scattering

Supplementary Figure 8 presents the changes in the hydrodynamic diameters (*D*_h_) of POPC and POPC:POPG (3:1) vesicles upon addition of Htr or Htr-M. Aliquots were added to POPC:POPG and POPC LUV dispersions leading to an increase of the respective *D*_h_ until a plateau was reached, followed by a decrease of the size of the supramolecular structures. In Tris-HCl buffer, pH 8.0, both peptides interact more strongly with the negatively charged vesicles, whereas the hydrodynamic diameter variation of POPC LUVs caused by the presence of Htr-M is negligible (data not shown). This latter observation is in agreement with the low association of Htr-M with zwitterionic lipid bilayers. The results obtained for Htr-M in the presence of DTT are very similar to the ones obtained for Htr-M indicating that the monomer does not undergoes dimerization during the experiments (Supplementary Figure 8).

### Antimicrobial assays

The antimicrobial activities of the monomer and dimer were assayed against Gram-positive (*S. aureus*), and Gram-negative bacteria (*P. aeruginosa* and *E. coli)*. Conventional antibiotics were used as positive controls. The Htr peptide showed consistently higher antimicrobial activities when compared to Htr-M exhibiting a minimal inhibitory concentration (MIC) of 11.6 μM, 1.5 μM and 23.2 μM for *S. aureus, E. coli* and *P. aeruginosa*, respectively. The monomer showed no antimicrobial activity against *P. aeruginosa*, while for *S. aureus* and *E. coli* the MICs were approximately four- and fifteen-fold higher when compared to Htr (Table 3).

## Discussion

Here we present for the first time the homodimeric peptide homotarsinin which exhibits significant antimicrobial activities. In aqueous phosphate buffer the homodimer exhibits a novel tridimensional structure, which has been characterized in atomic detail. Indeed, in aqueous medium chain-chain interactions within the homodimer result in a helical coiled coil conformation. These findings are in line with the heterodimeric antimicrobial peptide distinctin, in which inter-chain interactions were partially responsible for the higher structural stability of the dimer when compared to its individual chains in either aqueous or membrane environments^27^,^28^.

The solution NMR structure of Htr-M exhibits a highly amphiphilic character (Fig. 3) similar to other peptide antibiotics such as phylloseptins^24^, magainins^29^ and cecropins^30^,^31^. However, the hydrophobic face is interrupted by K10, which enhances the inter-chain contacts within the Htr homodimer by interacting with D5 and S9 of the opposite chain (Fig. 4c and e). The coiled coil structure of Htr is further stabilized by hydrophobic interactions, where the closely packed dimer assures that the hydrophobic residues are screened from the solvent. A more detailed comparison between the Htr-M and the Htr structures indicates that, in spite of the fact that the three helices extend over roughly the same residues, the chains of the dimer are slightly bent when compared to the monomer, a structural feature which is also endorsed by the presence of d_αN_(i, i + 2) NOE correlations for Htr but not for Htr-M^32^. Furthermore, by bringing the two chains into close proximity the disulfide bond plays an important role in maintaining the packing of the Htr coiled coil arrangement thereby positioning the hydrophobic residues and the cluster of polar Lys, Asp and Ser side chains for efficient inter-chain interactions. This arrangement leaves a solvent exposed surface of hydrophilic residues.

Notably, the presence of phosphate in the aqueous buffer strengthens the helical secondary structure of Htr, but not of Htr-M (Fig. 1), probably by simultaneously interacting with cationic side chains from both chains of the dimer. A smaller increase in the helical content is observed at higher concentrations of Tris probably due to the screening of repulsive forces between same charges. Interestingly, when a phosphate concentration of 100 μM and a peptide-to-phosphate ratio of 1:2 is reached (charges 3:1; Fig. 1), the helicity becomes maximal, suggesting that the bivalent phosphate ions are interacting with a selected set of cationic side chains. H-D exchange experiments are in agreement with phosphates being involved in the tight packing of the homodimer (Supplementary Figure 9)

When the homotarsinin structure in aqueous environments is compared to that of the heterodimer distinctin^27^, an interesting difference becomes evident. Whereas the coiled coiled structure of Htr efficiently shields the hydrophobic surface of the amphipathic helix from the aqueous buffer the side-by-side arrangement of the two chains in distinctin leaves such hydrophobic residues partially exposed. As a consequence distinctin forms a dimer of dimers in which hydrophobic side chain interactions are maximized in the inner part of the four-helix bundle^27^. Indeed, the heterodimeric structure of distinctin as well as oligomerisation of other sequences prevents the peptides from degradation by peptidases^27^,^33^,^34^. In the case of homotarsinin the densely packed coiled coil structure may be sufficient to protect the dimer from peptidases. Furthermore, the homotarsinin three-dimensional structure exposes positively charged residues (K, R and H) to the outer medium, which facilitate the peptide binding to the bacterial membrane surface. These results are in line with size exclusion chromatography experiments, which indicate that distincin elutes significantly faster than homotarsinin, despite their similar molecular masses, suggesting that Htr exists as a single dimer whereas distinctin is associated within a pair of dimers (Supplementary Figure 5).

The membrane interactions of homotarsinin were investigated by several biophysical techniques where the properties of the homodimer and of the monomer were compared to each other. As with many other polypeptides^25^,^35^,^36^ the presence of membranes enhances the propensity for amphipathic helical folds also for Htr-M and Htr where this secondary structure encompasses almost the full length of the peptide chains (Figs 1, 3 and 4).

When the membrane association of Htr-M was tested only weak interactions with POPC and moderate association with POPC:POPG (3:1) were observed (*K*_app_ = 326.2 L.mol^−1^). In contrast, the titrations of the homodimer with both LUVs showed significant peptide-lipid interactions, but again a higher affinity was observed for the negatively charged lipid bilayer. At 25 °C the affinity of Htr for POPC:POPG (3:1) LUVs is about six times higher than the affinity for POPC LUVs with *K*_app_ values of 7.0 × 10^3^ L.mol^−1^ and 1.2 × 10^3^ L.mol^−1^, respectively.

For both Htr-M and Htr significant exothermic enthalpies indicate that electrostatic lipid-peptide interactions are important for the membrane binding process, which is in line with results obtained from ITC, CD, and fluorescence experiments during the investigation of other linear cationic antimicrobial peptides, such as indolicidin and tritrpticins^37^,^38^. The hydrophilic surface of both peptides exposes a high density of overall cationic charges which help in the association with negatively charged membranes^39^. The apparent stoichiometric coefficients (*n*) suggest that membrane association saturates at lipid-to-peptide molar ratios of 10:1 and 17:1 for POPC and POPC:POPG (3:1) LUVs, respectively (Supplementary Table 1).

Notably, the interactions between the dimer and the zwitterionic vesicles are more pronounced than that of the monomer with POPC membranes indicating that the simultaneous action of two covalently linked chains facilitates peptide association to membranes, a process which is also paralleled by higher entropic contributions for the Htr dimer (−*T*Δ*S*° = −4180 cal/mol) in comparison with the Htr-M monomer (−*T*Δ*S*° = −1132 cal/mol).

A consistently smaller amount of carboxyfluorescein is released in the presence of Htr-M when compared to Htr and as expected the dye leakage rises with increasing concentrations of both monomer and dimer (Supplementary Figure 7). This data corroborates the proposed higher membrane-association of Htr discussed earlier. However, both peptides were more active in permeating the zwitterionic POPC when compared to negatively-charged POPC:POPG (3:1) membranes (Supplementary Figure 7) suggesting that although the negative charges increase binding they also trap the homotarsinin peptides in a topology less prone to disrupt the membrane^40^,^41^. Interestingly, in presence of phosphate buffer a lower percentage and slower kinetics of carboxyfluorescein release were observed for homotarsinin. Because phosphate buffer has been shown to stabilize the helical coiled coil structure of the dimeric peptide this observation suggests that the membrane insertion goes along with considerable changes in the tertiary fold similar to the rearrangements that have been monitored for distinctin upon membrane interaction^28^.

These results are also in line with DLS experiments, which show a biphasic behavior upon titration with Htr. Initially a pronounced increase in the LUV hydrodynamic diameter is observed, followed by a decrease at peptide-to-lipid ratios >0.1. This suggests that the peptide adsorption to the membranes is followed by vesicle lysis at higher peptide-to-lipid ratios (Supplementary Figure 8b)^42^. Notably, the peptide concentration dependence of the curves shown in Supplementary Figure 8 reflects the differences in association of Htr-M and Htr with the zwitterionic and the mixed membranes as observed by CD spectroscopy and ITC (Figs 1 and 5). Furthermore, for POPC:POPG LUVs in Tris-HCl buffer, the maximum *D*_h_ is observed at a molar ratio of approximately 0.1, whereas in the presence of phosphate buffer, known to stabilize the coiled coil structure (Figs 1 and 4), the maximum occurs at a molar ratio about twice as high (~0.2).

These stronger membrane interactions and the higher membrane disruptive properties of Htr compared to Htr-M are in excellent agreement with the significantly higher activities of Htr against the three bacterial strains tested. While the activity of Htr-M against *P. aeruginosa* was not determined, Htr presents an activity even stronger than the control drugs (Table 3). In the assays against *S. aureus* and *E. coli* Htr showed activities 4 and 16 times higher than Htr-M, respectively. Therefore, the biological assays, combined with ITC and DLS investigations, clearly indicate that the disulfide bond linking the two individual chains is a very important structural element for the membrane and antimicrobial activities of homotarsinin. Besides, in analogy to the compact four helix bundle structure of distinctin^27^, it is believed that the coiled coil arrangement of the homodimer protects homotarsinin against degradation by peptidases and that membrane interactions are concomitant with considerable structural rearrangements. Therefore, by comparing the structural and biophysical data as well as the activities of Htr in relation to its monomer it becomes obvious that the concerted action of the two chains plays a key role in the membrane binding and perforating process.

## Methods

### Peptide synthesis, purification and characterization

The homotarsinin monomer (Htr-M) and its derivative [C23S]Htr-M were obtained by solid phase synthesis, using the Fmoc (9-fluorenylmethyloxycarbonyl) strategy^43^. These linear peptides were synthesized with an amidated carboxy-terminus by using a Fmoc-Rink Amide Polystyrene Resin from Iris Biotech GmbH (Marktredwitz, Germany). All of the amino acid derivatives were from Iris Biotech GmbH. The homodimer (Htr) was prepared thereafter by air oxidation of the monomer in aqueous buffer according to Monera and co-workers^44^. The purified monomer was incubated at the concentration of 10 mg.mL^−1^ in 10 mM Tris-HCl, pH 8.5. The solution was left stirring and the reaction was monitored by RP-HPLC at room temperature for 72 h. After this time interval the stirring was stopped and the solution was neutralized with dilute acetic acid, lyophilized and then purified by RP-HPLC, using a VYDAC^®^ 218TP semi-preparative reversed-phase column (10 mm × 250 mm C18, Grace Corporate, Columbia, MD) equilibrated with 0.1% aqueous TFA and eluted with a linear gradient of acetonitrile in 0.08% TFA. A flow of 2.0 mL.min^−1^ was employed and the peptide detected at 214 nm. The purified products of Htr-M, [C23S]Htr-M and Htr were characterized through MALDI-TOF mass spectrometry using a MicroTOF apparatus (Bruker Daltonics; Billerica, MA).

### Vesicle preparation

The lipid vesicles were prepared using standard procedures^45^. The phospholipids 1-palmitoyl-2-oleoyl-phosphatidylcholine (POPC) and 1-palmitoyl-2-oleoyl-phosphatidylglycerol (POPG) were purchased from Avanti Polar Lipids (Birmingham, AL). The correct amounts of POPC or POPC:POPG (3:1 mole:mole) were dissolved and mixed in dichloromethane. The solvent was removed with a rotary evaporator; the resulting thin film dried in vacuum for at least one hour to remove the residual solvent and then hydrated with a 10 mM Tris-HCl buffer solution (pH 8.0) or 10 mM phosphate buffer (pH 7.0). Large unilamelar vesicles (LUVs) were obtained by extruding the suspension eleven times through two 100 nm polycarbonate membranes in a LiposoFast^®^ extrusion system (Avestin Inc., Ottawa, ON, Canada). Entrapment of carboxyfluorescein (CF) in 1 mM LUVs was carried out by hydrating a thin film with a buffer containing 10 mM Tris–HCl, pH 8.0, and 10 mM CF. Free CF was removed by passing 1 mL of the extruded LUVs through a Sephadex-G25 column (1.2 cm × 20 cm) and elution with 10 mM Tris–HCl, pH 8.0, containing 200 mM NaCl. The LUVs were collected at the initial volume (V_o_) and the PC content of the eluted LUVs was determined by phosphorous assay as described in literature^46^.

### Circular Dichroism Spectroscopy

CD spectroscopic analyses of the Htr, Htr-M and [C23S]Htr-M secondary structures were performed in aqueous buffer solutions (Tris-HCl, pH 8.0, and phosphate buffer, pH 7.0), in TFE:H_2_O solutions and in the presence of POPC and POPC:POPG (3:1, mole/mole) phospholipid vesicles. CD spectra were recorded at 20 °C on a Jasco-715 spectropolarimeter (Jasco, Tokyo, Japan) using a 1.0 mm path length rectangular quartz cuvette (Uvonic Instruments, Plainview, NY). All spectra were recorded with eight scans from 260 to 190 nm using a 1.0 nm spectral bandwidth, 0.2 nm step resolution, 100 nm.min^−1^ scan speed, and 4 s response time. Similar experiments with the respective blank solutions were also carried out in order to allow for background subtraction. Homodimer, monomer and [C23S]Htr-M concentrations in the samples were 40 μM, 45 μM and 45 μM, respectively. The spectra were analyzed using the CDPro software including CONTIN, SELCON and CDSSTR methods for estimation of peptide secondary structure^47^.

### Dynamic light scattering

DLS experiments were performed at 25 °C in a Malvern Zetasizernano ZS^®^ particle analyzer (Malvern Instrument Ltd, Worcestershire, UK). Measurements of the average hydrodynamic diameter of pure and peptide-bound LUVs were done using a polyethylene square cell. The solutions were subjected to scattering by a monochromatic light (10 mW He-Ne laser, wavelength 632.4 nm) and the scattered light intensity was measured at an angle of 90°. The hydrodynamic diameters are the average of five independent measurements, with twenty counts per measurement. A solution of 1.0 mM LUVs in 10 mM Tris-HCl buffer, pH 8.0, or in 10 mM phosphate buffer, pH 7.0, both containing 200 mM NaCl, was titrated with 1 mM peptide prepared in the same buffer. Titration was conducted by addition of fifty aliquots (10 μL each) of the titrating solution.

### Isothermal titration calorimetry

Isothermal titration calorimetry (ITC) experiments were performed at 25 °C with a high-precision VP-ITC Microcalorimeter^®^ (Malvern Instrument Ltd, Worcestershire, UK) for 100 μM peptide solutions in 10 mM Tris-HCl, pH 8.0, containing 200 mM NaCl or in 10 mM phosphate buffer, pH 7.0, containing 200 mM NaCl. The ITC equipment was electrically and chemically calibrated before performing the experiments^48^. All solutions and buffers employed in the experiments were filtered and degassed under reduced pressure (140 mbar) for eight minutes. The peptide solutions in the calorimeter cell were titrated with fifty-one successive injections of 20 mM POPC or POPC:POPG (3:1, mole:mole) LUVs (the first 1 μL injection was discarded in order to eliminate diffusion effects of the material from syringe to calorimeter cell and this injection was followed by fifty injections of 5 μL). Injection times of 2 s with intervals of 240 s have been used in the experiment. A similar titration was performed with buffer in the calorimeter cell using the same lipid suspensions to determine the corresponding heats of dilution, which were then subtracted from the heats determined in the corresponding peptide-to-lipid binding experiments. To measure the heat produced by the peptide-to-lipid interactions, each peak of the calorimetric curve was integrated following standard procedures as described by Wieprecht and Seelig^39^. The total lipid concentration was used to estimate the degree of membrane association as well as in the determination of the thermodynamic binding parameters^49^. The raw data were analyzed with the software Microcal Origin 5.0 (OriginLab Corporation, Northampton, MA) for ITC, supplied together with the microcalorimeter.

### Dye leakage measurements

Aliquots of LUVs (15 μL) were added to a standard profile polystyrene plates (dimensions 128 × 86 × 14.5 mm) for fluorescence measurements containing 285 μL of the same buffer used for the Sephadex-G25 column elution. The increase of CF fluorescence as a function of time at 25 °C was continuously recorded in Spectra Max® Paradigm (Molecular Devices, LLC, Sunnyvale, CA) using λ_ex_ = 490 nm and λ_em_ = 512 nm. At the end of each experiment, total CF fluorescence was determined by the addition of 20 μL of 10% (w/v) Triton X-100. The percentage of CF leakage (%CF), was determined as previously described in literature^50^.

### Antibacterial Assays

The antimicrobial activities of Htr-M and Htr were investigated against three bacterial strains, *Pseudomonas aeruginosa* ATCC 27853, *Staphylococcus aureus* ATCC 43300 and *Escherichia coli* ATCC 25992, all from American Type Culture Collection (Manassas, VA). The microorganisms were cultured at the Laboratório de Ecologia e Fisologia de Microorganismos, Departamento de Microbiologia, ICB-UFMG, Belo Horizonte, Brazil. The microorganisms in stationary culture at 37 °C were transferred to Müller-Hinton liquid medium, according to the National Committee for Clinical Laboratory Standards (NCCLS) to perform the assays^51^. The highest peptide concentration used for the assays were 23.5 μM against *E. coli* and *P. aeruginosa* and 47.0 μM against *S. aureus* in an initial inoculum of 2.5 × 10^5^ cfu.mL^−1^ (colony-forming units.mL^−1^). The peptide was diluted up to eight-fold in Müller-Hinton liquid broth by serial dilution. The final volume was 100 μL per well, 50 μL of the peptide and 50 μL of the inoculum. The experiment was carried out in stationary culture at 37 °C, and the spectrophotometer readings were performed 12 h after incubation. The minimal inhibitory concentration was determined based on three independent measurements, using the optical density parameter (A_595_ nm). Conventional antibiotics (ampicillin and chloramphenicol) had their minimum inhibitory concentrations determined against the three experimental bacterial strains. All assays were performed in triplicate.

### Nuclear Magnetic Resonance Spectroscopy

Htr-M was dissolved in a mixture of H_2_O/TFE-*d*_2_ (70:30, v:v) at 4 mM concentration. The pH was adjusted to 7.0 with 20 mM aqueous phosphate buffer. A sample containing Htr at 1 mM was prepared by dissolving the peptide in water containing 5% of TFE-*d*_2_ (v:v) and 5 μM 2,2-dimethyl-2-silapentane sulfonate (DSS as internal reference). The pH was adjusted to 7.0 with 20 mM aqueous phosphate buffer. ^1^H NMR spectra for H-D exchange experiment were recorded at 25 °C on a 600 MHz Bruker Avance III spectrometer using 1 mM of dimer in D_2_O and in a D_2_O containing 5% of TFE-*d*_3_ (v:v) pH 7.0 phosphate buffer solution.

Two-dimensional (2D) NMR spectra of Htr-M were recorded on a Bruker *AVANCE III* 600 NMR spectrometer operating at 600.043 MHz and the spectra of Htr were recorded on a Bruker *AVANCE III* 800 NMR spectrometer operating at 800.118 MHz (Brazilian National NMR Center – Rio de Janeiro). Triple-resonance (^1^H/^13^C/^15^N) gradient probes (5 mm sample diameter) were used in all of the experiments. Water suppression was achieved by using pre-saturation techniques. The sample temperature for all of the experiments was maintained at 20 °C. TOCSY spectra were acquired using the MLEV-17^52^ pulse sequence with a spin-lock time of 80 ms. The spectral width was 9615 Hz for the Htr sample and 6900 Hz for Htr-M sample, 512 *t*_*1*_ increments were collected with 16 transients of 4096 points. NOESY spectra^53^ were acquired using mixing times of 80, 100, 120, 140, 200 and 400 ms for the Htr-M sample and 80, 100, 120, 140, 160, 200 ms for the Htr sample. The spectral width was 9615 Hz for the Htr sample and 6900 Hz for the Htr-M sample, 512 *t*_*1*_ increments were collected with 16 transients of 4096 points for each FID. ^1^H-^13^C HSQC spectra were acquired for Htr with F1 and F2 spectral widths of 72010 Hz and 9615 Hz, respectively, and for Htr-M with F1 and F2 spectral widths of 60004 Hz and 6900 Hz. 128*t*_*1*_ increments were collected with 128 transients of 1024 points. The experiments were acquired in an edited mode in such a way that CH and CH_3_ correlations show positive phase and CH_2_ correlations show negative phase^53^,^54^. ^1^H-^15^N HSQC spectra were acquired for Htr with F1 and F2 spectral widths of 27160 Hz and 9615 Hz respectively, and for Htr-M with F1 and F2 spectral widths of 21280 Hz and 6900 Hz respectively. 80 *t*_*1*_ increments were collected with 400 transients of 1024 points for each free induction decay^54^. All NMR spectra were processed using NMRPipe^55^.

### NMR data analysis and structure calculations

^1^H resonance assignments were performed by simultaneously analyzing 2D ^1^H-^1^H TOCSY and NOESY spectra, as elaborated by Wüthrich^32^, using the NMRView software^56^. NOE intensities obtained at mixing times of 140 ms (Htr-M) and 100 ms (Htr) were converted into semi-quantitative distance restrains by using calibrations by Hybert and co-workers^57^. The upper limits of the distances thus obtained were 2.8, 3.4 and 5.0 Å (for strong, medium, and weak NOEs, respectively). Angular restrains have been obtained from analysis of C_α_, H_α_, C_β_, N and H_N_ chemical shifts with the program TALOS+^58^. Structure calculations were performed using the Xplor-NIH software, version 2.14^59^. Starting with an extended conformation 200 structures were generated using a simulated annealing protocol. This was followed by 20000 steps of simulated annealing at 1000 K and a subsequent decrease in temperature in 15000 steps in the first slow-cool annealing stage. The twenty lowest energy structures were refined by using a *sa_new.inp* protocol and the stereochemical quality was analyzed by PROCHECK-NMR^22^. The display, analysis, and manipulation of the three-dimensional structures were performed with the program MOLMOL^60^.

### Size exclusion chromatography

The assays were performed for 2 mg/mL aqueous solutions of the peptides homotarsinin and distinctin at room temperature, using a 1.5 × 50 cm Dextrana Superdex Peptide Tric. 10/300 GL (GE Healthcare, Little Chalfont, UK) coupled to a Varian Pro-Star 330 chromatograph (Varian Inc, Palo Alto, CA) at a flow rate of 0.3 mL/min. A 20 mM aqueous phosphate buffer solution, pH 7.0, containing NaCl at 0.3 M was used as mobile phase.

## Additional Information

**How to cite this article**: Verly, R. M. *et al*. Structure and membrane interactions of the homodimeric antibiotic peptide homotarsinin. *Sci. Rep.* **7**, 40854; doi: 10.1038/srep40854 (2017).

**Publisher's note:** Springer Nature remains neutral with regard to jurisdictional claims in published maps and institutional affiliations.

## Supplementary Material

Supplementary Information

## Figures and Tables

**Table 1:** Helical contents obtained from CD spectroscopy for Htr-M and Htr in the following media: TFE/H_2_O mixtures, POPC LUVs, POPC:POPG (3:1 mole:mole) LUVs, Tris-HCl buffer, pH 8.0, and phosphate buffer, pH 7.0.

TFE/H_2_O % (v/v)	percentage of α-helix		POPC LUVs (μM)	percentage of α-helix	
	Htr-M	Htr		Htr-M	Htr
0	8(3)	18(4)	0	6(5)	17(6)
10	25(4)	55(5)	100	8(4)	20(5)
20	32(3)	66(4)	200	16(6)	40(3)
30	49(4)	69(3)	300	24(4)	48(4)
60	52(3)	73(4)	500	30(5)	50(3)
**Buffer (μM)**	**Htr-M Tris-HCl/phosp.**	**Htr Tris-HCl/phosp.**	**POPC:POPG (3:1) LUVs (μM)**	**Htr-M**	**Htr**
0	6(3)/7(3)	12(4)/14(5)	0	9(3)	17(5)
10	9(4)/9(3)	11(5)/13(4)	100	15(3)	25(4)
20	10(2)/12(3)	13(5)/18(6)	200	19(5)	47(5)
30	9(3)/15(4)	15(5)/27(4)	300	38(4)	59(4)
50	8(4)/10(3)	17(4)/45(2)	500	45(7)	65(2)

**Values determined as the average of CONTIN, SELCON and CDSSTR methods (followed in parentheses by the standard error of the mean).

**Table 2:** NMR and refinement statistics for structures of 4.0 mM Htr-M in TFE-d2:H2O (30:70) and 1.0 mM Htr in 20 mM phosphate buffer, pH 7.0 containing 5% TFE-d2.

	Htr-M	Htr
**NMR distance and dihedral constraints**	481	721
Distance constraints		
Total NOE		
Intra-residue	300	451
Inter-residue		
Sequential (|*i* − *j*| = 1)	103	141
Medium-range (|*i* − *j*| <4)	78	114
Long-range (|*i* − *j*| >5)	0	15
Total dihedral angle restraints	39	70
**Structure statistics**		
Ramachandran analysis		
Residues in most favored regions	96.9%	94.6%
Residues in additional allowed regions	2.4%	4.8%
Residues in generously allowed regions	0.2%	0.6%
Residues in disallowed regions	0.5%	0.0%
Average pairwise r.m.s. deviation** (Å)		
Heavy	1.58 Å	1.20 Å
Backbone	0.89 Å	0.98 Å

^**^Pairwise r.m.s. deviation was calculated among 20 refined structures.

**Table 3:** Minimal inhibitory concentrations determined for Htr-M and Htr in the presence of ATCC bacteria.

Bacteria (ATCC strains)	Gram	MIC (μM)				
		Htr-M	Htr	DS01	Ampicillin	Chloramphenicol
*Staphylococcus aureus* ATCC 29313	Positive	46.5	11.6 (23.2)*	26.5	<11	>150
*Escherichia coli* ATCC 25922	Negative	23.2	1.5 (3.0)*	6.6	46	25
*Pseudomonas aeruginosa* ATCC 27853	Negative	>150	23.2 (46.4)*	NT	25	25

^*^MIC values per moles of monomer chain.

NT – not tested, DS 01 = antimicrobial peptide control.
