# Supplementary material for: Structure and membrane interactions of the homodimeric antibiotic peptide homotarsinin
Source: Sci Rep. 2017 Jan 19;7:40854. doi: 10.1038/srep40854 (PMC5244374; doi:10.1038/srep40854)
Supplement: Supplementary Information [file srep40854-s1.pdf]

SUPPLEMENTARY INFORMATION

Structure and membrane interactions of the homodimeric antibiotic peptide homotarsinin

Rodrigo M. Verly, Jarbas M. Resende, Eduardo F. C. Junior, Mariana T. Q. de Magalhães, Carlos F. C. R. Guimarães, Victor H. O. Munhoz, Marcelo P. Bemquerer, Fábio C. L. Almeida, Marcelo M. Santoro†, Dorila Piló-Veloso, Burkhard Bechinger*

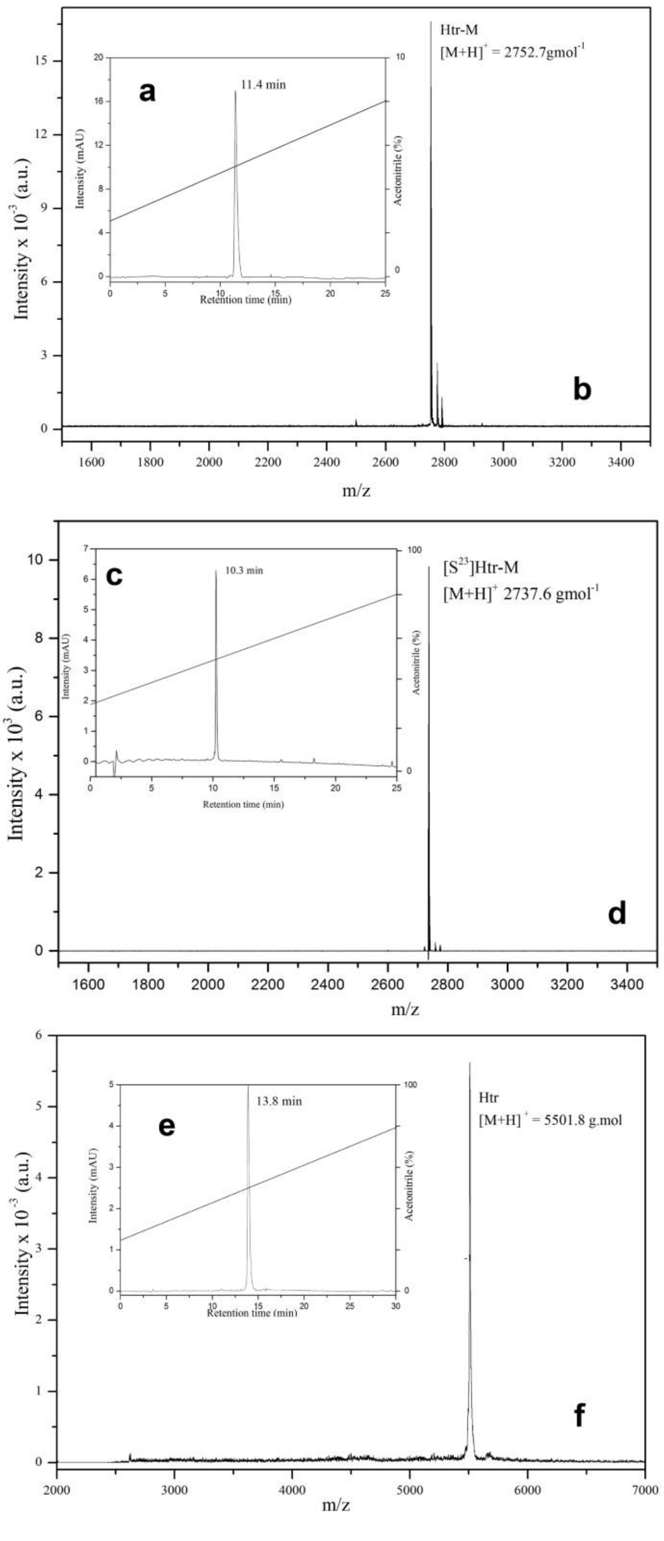

Supplementary Figure 1

Characterization of synthetic Htr-M, [C23S]Htr-M and Htr.

Reversed-phase HPLC chromatogram of (**a**) Htr-M, (**c**) [C23S]Htr-M and (**e**) Htr peptides after synthesis and purification using a Vydac column (10 mm x 250 mm C18) equilibrated with 0.1% aqueous TFA and eluted by a linear gradient of acetonitrile in 0.08% TFA. A flow rate of 1.0 mL.min^{-1} was employed and the peptide detected at 214 nm. The MALDI-TOF spectrum of purified (**b**) Htr-M, (**d**) [C23S]Htr-M and (**f**) Htr. The molecular masses of Htr-M, [C23S]Htr-M and Htr were measured at 2752.7 Da, 2737.6 Da and 5501.8 Da respectively, obtained in a Bruker Autoflex instrument (Bilerica, MA).

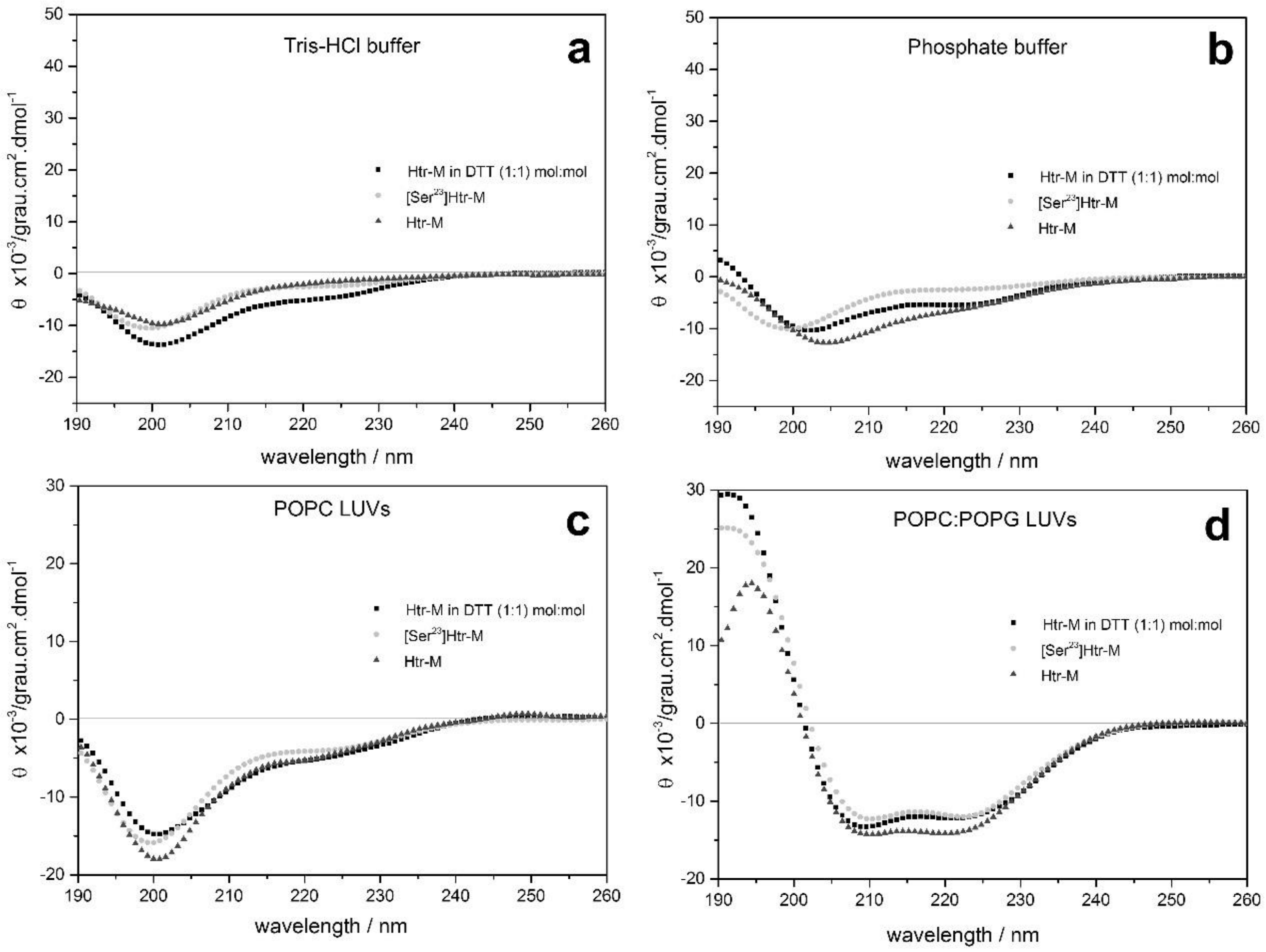

Supplementary Figure 2

CD spectra for Htr-M, Htr-M in the presence of DDT and [C23S]Htr-M.

CD spectra of 49 μM Htr-M (filled triangle), 50 μM Htr-M in DTT (1:1, mole:mole) (filled square) and 50 μM [C23S]Htr-M (filled circle). (**a,b**) Spectra recorded for the peptides in 100 μM Tris–HCl, pH 8.0, buffer solutions and 100 μM phosphate buffer, pH 7.0. (**c,d**) Spectra recorded for the peptides in the presence of 500 μM phospholipid POPC LUVs and POPC:POPG (3:1 mole:mole) LUVs.

CD spectra obtained for Htr-M, Htr-M in the presence of DDT and [C23S]Htr-M in aqueous phosphate buffer, pH 7.0 and Tris buffer, pH 8.0, as well as in the presence of phospholipid vesicles are very similar to each other, which indicates that dimerization does not take place in either aqueous or membrane environments.

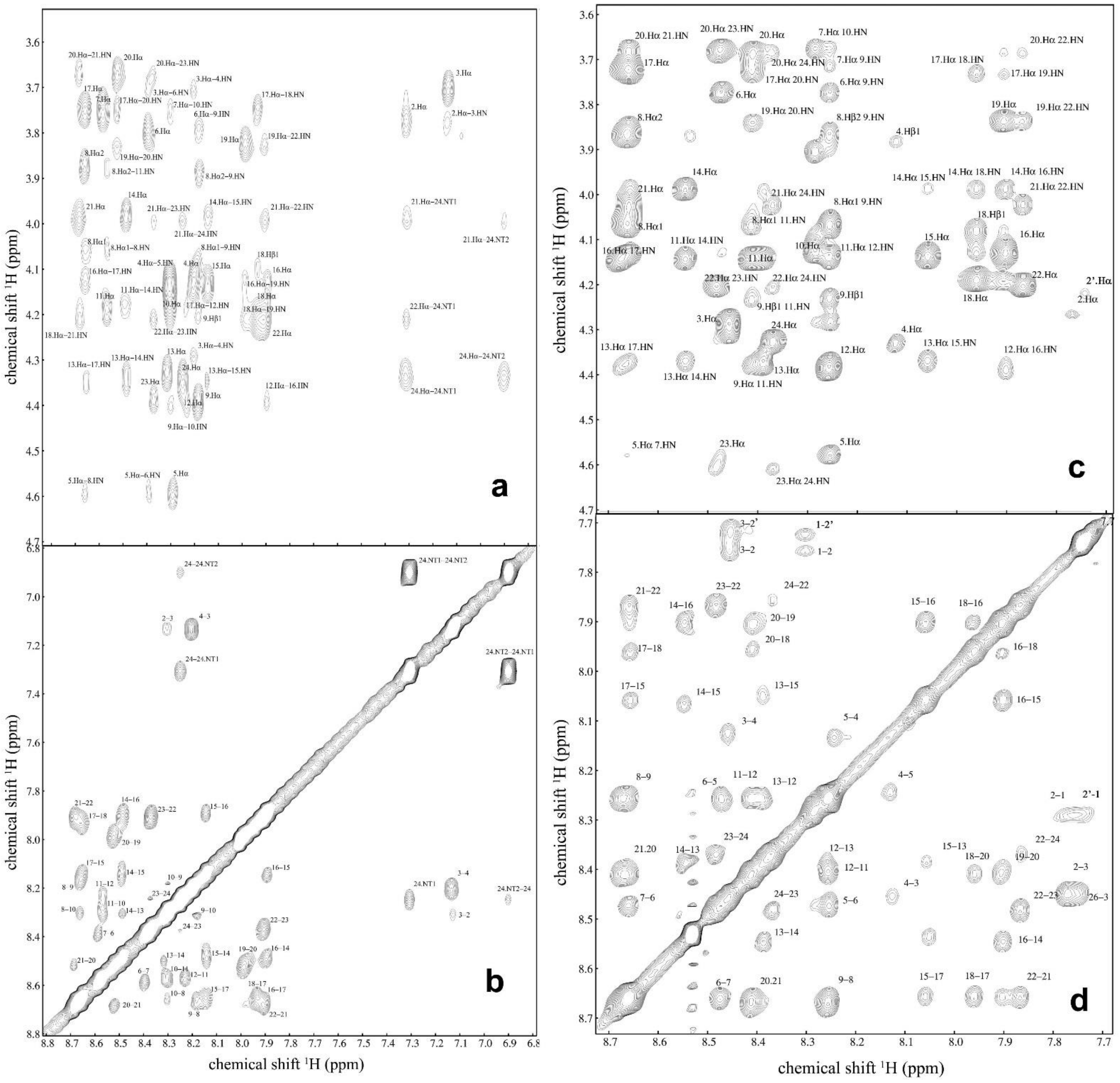

Supplementary Figure 3

Proton cross-peak regions of the NOESY

(**a**) Fingerprint (H_N-H_α correlations) and (**b**) amide proton cross-peak regions of the NOESY spectrum of 4 mM Htr-M in TFE-d_2/H_2O 30/70 (v/v). (**c**) Fingerprint (H_N-H_α correlations) and (**d**) amide proton cross-peak regions of the NOESY spectrum of 1 mM Htr in 20 mM aqueous phosphate buffer, pH 7.0, containing 5% of TFE-d_2 (v/v).

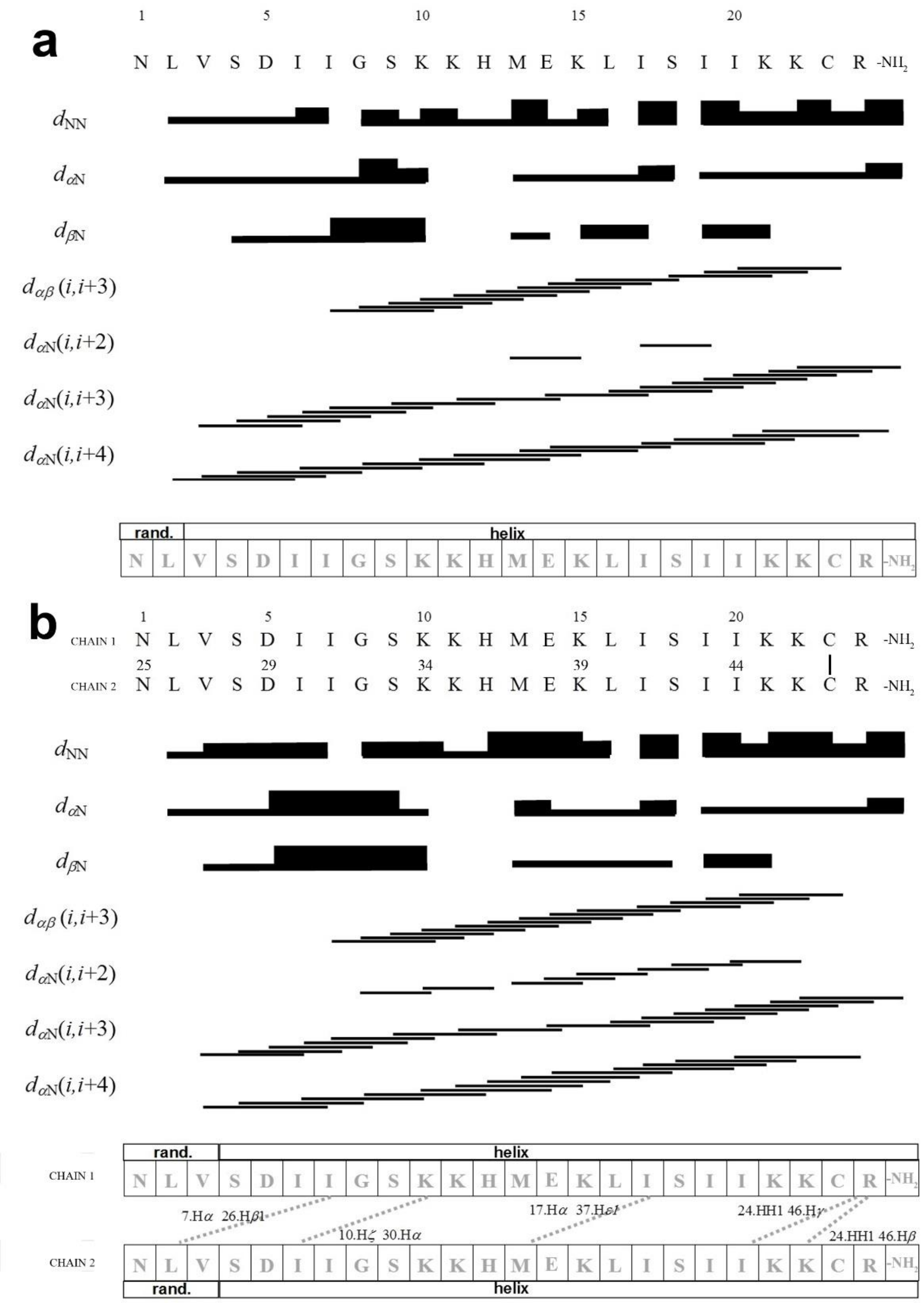

Supplementary Figure 4

Graphical representation of NOE cross peaks

(**a**) 4 mM Htr-M in H_2O/ TFE-*d*2 70/30 (v/v) and. (**b**) 1 mM Htr in 20 mM aqueous phosphate buffer, pH 7.0, containing 5% of TFE-*d*2, (v/v). The d_{NN}, $d_{\alpha N}$, and $d_{\beta N}$ are sequential NOE correlations, where thin, medium and thick bars represent weak, medium and strong NOEs, respectively. The $d_{\alpha N}$(i,i+3), $d_{\alpha\beta}$(i,i+3), and $d_{\alpha N}$(i,i+4) represent medium range correlations which are characteristic of α-helical secondary structures. The outlines of the helical conformation are also indicated. Long-range NOE correlations are presented for Htr. -NH_2 represents the carboxyamide terminus.

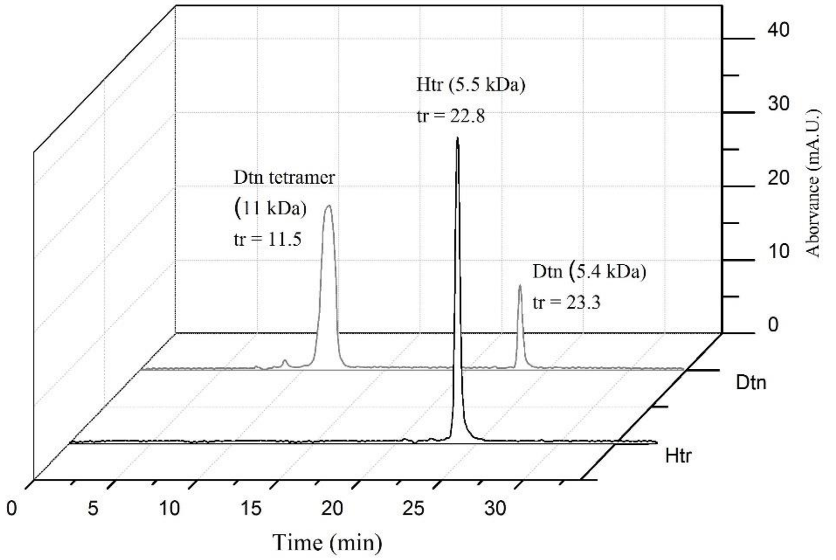

Supplementary Figure 5

Size-exclusion chromatography of homotarsinin and distinctin at room temperature. A Dextrana Superdex Column (1.5 × 50 cm Peptide Tric. 10/300 GL) was equilibrated with at a 20 mM aqueous phosphate buffer, pH 7.0, solution containing NaCl at 0.3 M. A flow rate of 0.3 mL/min was employed and the peptide was detected at 214 nm.

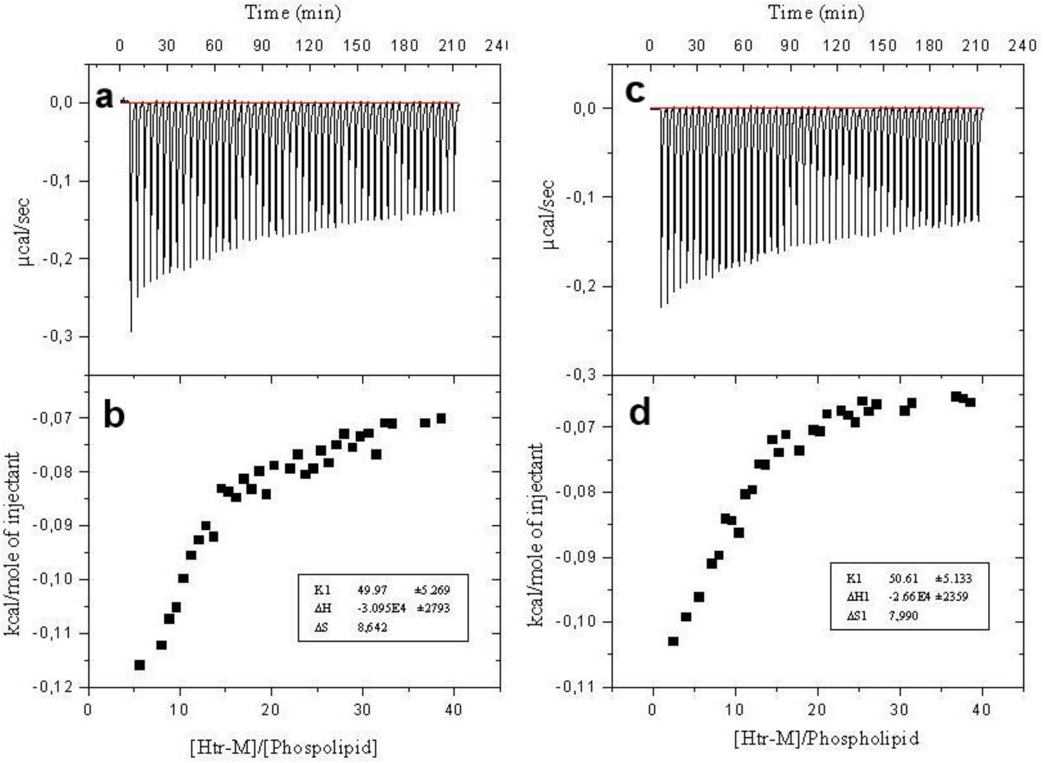

Supplementary Figure 6

Isothermal titration calorimetry of (**a,b**) 100 µM Htr-M and (**c,d**) 100 µM [C23S]Htr-M in 10 mM Tris–HCl, pH 8.0, containing 200 mM NaCl with POPC:POPG (3:1) LUVs (20 mM stock solutions in 10 mM Tris–HCl, pH 8.0 containing 200 mM NaCl). Upper: The heat flow for each peptide injection as a function of time (raw data). Down: The enthalpy as a function of the peptide-to-phopholipid molar ratio. The baseline was corrected and the heat of dilution subtracted from each experimental point.

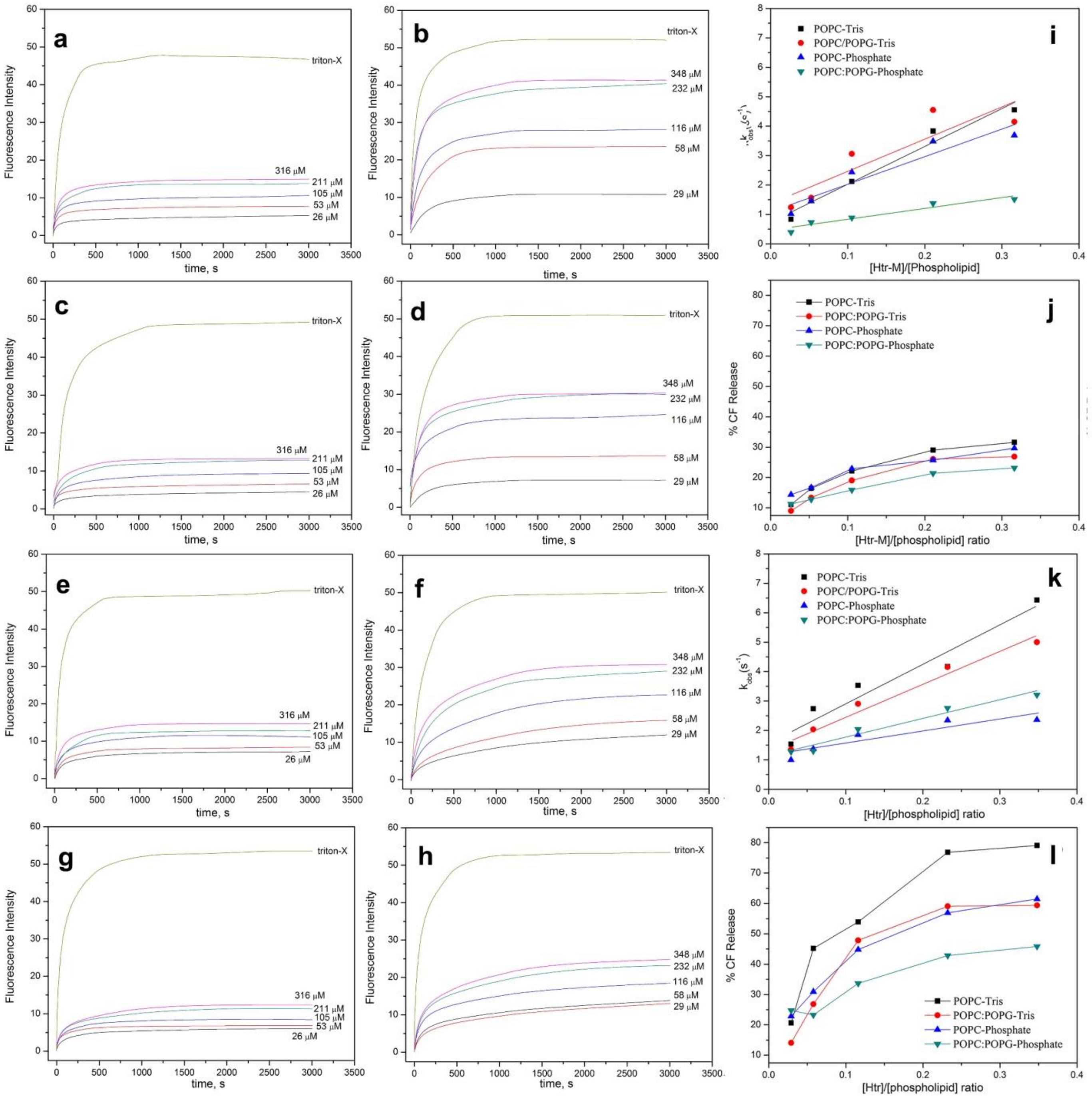

Supplementary Figure 7

Kinetics of CF release and observed rate constants, k_{obs}

(**a,c,e,g**) Kinetics of CF release from LUVs in presence of Htr-M and (**b,d,f,h**) Htr. (**a,b**) POPC-LUVs suspended in Tris-HCl buffer, pH 8.0, (**c,d**) POPC:POPG (3:1) LUVs suspended in Tris-HCl buffer, pH 8.0, (**e,f**) POPC LUVs suspended in phosphate buffer, pH 7.0 and (**g,h**) POPC:POPG (3:1) LUVs suspended in phosphate buffer, pH 7.0. Observed rate constants, k_{obs}, of CF release as a function of (**i**) [Htr-M]/[Phospholipid] and (**j**) [Htr]/[Phospholipid] molar ratio. Percentage of CF release as function of (**k**) [Htr-M]/[Phospholipid] and (**l**) [Htr]/[Phospholipid] molar ratio. Measurements were done at 2500 s. Samples were added to a microplates containing 10 mM Tris–HCl buffer, pH 8.0 and 200 mM NaCl or 10 mM phosphate buffer, pH 7.0 and 200 mM NaCl. Fluorescence emission was measured using λ_{ex} = 490 and λ_{em} = 512 nm.

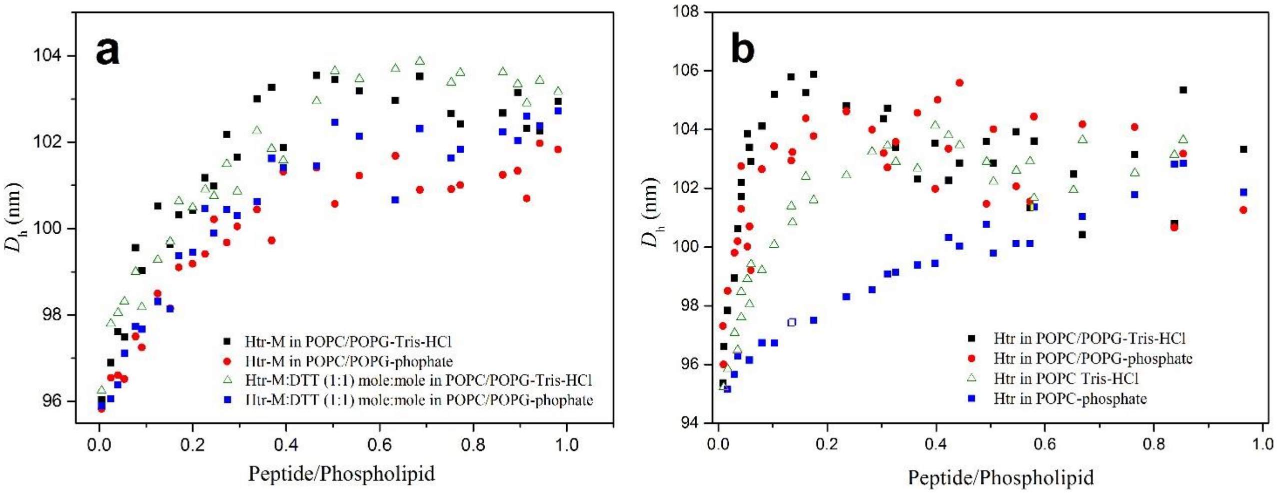

Supplementary Figure 8

Hydrodynamic diameter of LUVs as a function of the peptide/phospholipid ratio.

(**a**) [peptide/phospholipid] molar ratio of the Htr-M (black squares) and Htr-M in presence of DTT (1:1) (green open triangle) titrated with POPC:POPG LUVs in a 10 mM Tris–HCl, pH 8.0 (200 mM NaCl) and Htr-M (red circles) and Htr-M in presence of DTT (1:1) (blue squares) titrated with a POPC:POPG LUVs in 10 mM phosphate buffer, pH 7.0 (200 mM NaCl). (**b**) [Htr/phospholipid] molar ratio of the peptide titrated with POPC:POPG LUVs in a 10 mM Tris–HCl, pH 8.0 (200 mM NaCl) (black squares), titrated with POPC:POPG LUVs in a 10 mM phosphate buffer, pH 7.0 (200 mM NaCl) (red circle), titrated with POPC LUVs in a 10 mM Tris–HCl buffer, pH 8.0 (200 mM NaCl) solution (green triangles) and titrated with a POPC LUVs in a 10 mM phosphate buffer, pH 7.0 (200 mM NaCl) solution (blue triangles).

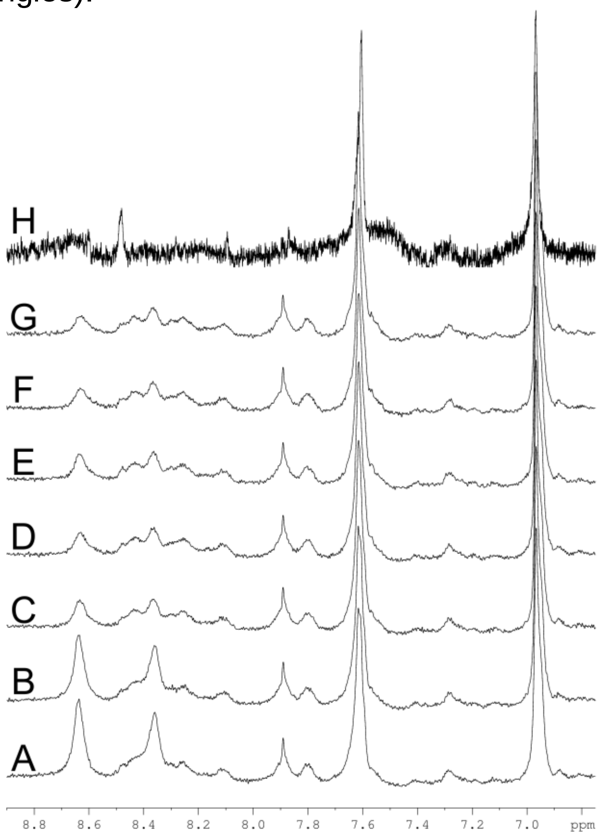

Supplementary Figure 9

Amide region of the ^{1}H NMR spectra of Htr in (A-G) in a D_2O containing 5% of TFE-d_3 (v:v) and phosphate buffer solution, pH 7.0, and in (H) D_2O. (A) and (H) were obtained 10 min after sample preparation, (B) after 1 h, (D) after 2 h, (D) after 4 h, (E) after 8 h, (F) after 12 h and (G) after 24 h. The spectra were recorded at 25 ^oC on a 600 MHz Bruker Avance III spectrometer.

In order to investigate the structural stability of the coiled-coil conformation of Htr in the presence of phosphate buffer we have recorded ^{1}H NMR spectra of the dimer in D_2O and in a D_2O containing 5% of TFE-d_3 (v:v) pH 7.0 phosphate buffer solution. Practically all of the amide hydrogen resonances have disappeared in the spectrum recorded in D_2O (panel H) 10 min after the sample preparation. On the other hand, several amidic hydrogen resonances remained in the spectrum recorded for the peptide in a D_2O containing 5% of TFE-d_3 (v:v) and phosphate buffer solution, pH 7.0, even 24 hours after sample preparation (panels A-G). These results are in line with CD spectroscopy, which indicates that the presence of phosphate buffer is essential to assure the helical conformation of the peptide in aqueous medium (Figure 1b). The obtained results also indicate that the two helical segments are tightly packed within the homodimer coiled-coil structure.

Supplementary Table 1

Thermodynamic parameters from ITC experiments where suspensions of 20 mM POPC or POPC:POPG (3:1) LUVs, respectively, were titrated into 100 μM Htr (10 mM Tris–HCl, pH 8.0, 200 mM NaCl at 298 K).

**LUVs**	***n***	K_{app}(L.mol^{-1})	ΔG^o(cal.mol^{-1})	ΔH^o(cal.mol^{-1})	ΔS^o(cal.mol^{-1}.K^{-1})
Htr POPC	9.6 ± 0.3	1274 ± 108	-4234	-694 ± 22	11.87
Htr POPC/POPG (Per monomer chain)	17.0 ± 0.2	7030 ± 198	-5243	-1063 ± 64	14.02
